# Does Religiosity in the Transition to Adulthood Predict Filial Norms in Midlife?

**DOI:** 10.1093/geroni/igab046.1525

**Published:** 2021-12-17

**Authors:** Jeung Hyun Kim, Woosang Hwang, Maria Brown, Merril Silverstein

**Affiliations:** 1 Syracuse University, Syracuse, New York, United States; 2 Syracuse University, Syracuse, New York, New York, United States

## Abstract

Objective: This study aims to identify multiple dimensions of religiosity among young adults at the beginning and end of the transition to adulthood, and describe how transition patterns of religiosity in early adulthood are associated with filial elder-care norms in midlife. Background: There is a broad consensus that religiosity is multidimensional in nature, but less is known regarding transitions in multiple dimensions of religiosity from early to middle adulthood and predicted filial eldercare norms as a function of those religiosity transitions. Methods: The sample consisted of 368 young adults participating in the Longitudinal Study of Generations in 2000 (mean age = 23 years) and 2016 waves. We conducted a latent class and latent transition analyses to address our aims. Results: We identified three religious latent classes among young adults in both 2000 and 2016 waves: strongly religious, weakly religious, and doctrinally religious. Staying strongly religious young adults between 2000 to 2016 waves reported higher filial elder-care norms in the 2016 Wave than those who were in staying weakly religious, staying doctrinally religious, and decreasing religiosity transition patterns between 2000 to 2016 waves. Conclusion: Our findings suggest that religiosity is still an important value for young adults shaping their intergenerational relationships with their aging parents. Keywords: religiosity, filial eldercare norms, young adults, transition to adulthood

